# Histological and quantitative polymerase chain reaction-based analysis of Buruli ulcer using mapping biopsy method

**DOI:** 10.1371/journal.pntd.0008051

**Published:** 2020-06-22

**Authors:** Toshifumi Takahashi, Miho Kabuto, Gen Nakanishi, Toshihiro Tanaka, Noriki Fujimoto

**Affiliations:** Department of Dermatology, Shiga University of Medical Science, Setatsukinowa, Otsu, Shiga, Japan; Faculty of Science, Ain Shams University (ASU), EGYPT

## Abstract

**Background:**

In Japan, Buruli ulcer cases are often advanced, requiring surgical treatment. However, extensive debridement is often difficult because of cosmetic and functional sequelae. Moreover, the lesions are complicated and composed of edematous erythema, necrotic ulcer, and erythematous skin lesions caused by a paradoxical reaction, which also make it difficult to perform adequate debridement.

**Methodology/Principal findings:**

We performed quantitative polymerase chain reaction (PCR) analysis for IS*2404* using 29 samples taken from mapping biopsy. We evaluated the relationship among mycobacterial burden, histopathological findings, and clinical outcomes using 83 tissue samples taken from mapping biopsy and debrided Buruli ulcer. On quantitative PCR, the Cp values of IS*2404* amplification were substantially different in each site. The major histological findings could be divided into massive subcutaneous necrosis with scant inflammatory cell infiltration and dense inflammatory cell infiltration. Of the 84 sites, 34 were subjected to repeated histological evaluations. In these sites, histological necrosis did not disappear over time despite standard antibiotic treatment. In contrast, the ulcers were cured and no recurrences were observed without resecting the 11 biopsied sites that lacked histological necrosis. Although quantitative PCR revealed that a lower Cp value of IS*2404* was associated with histological massive necrosis, sites that showed lower Cp values clinically did not always need debridement.

**Conclusion/Significance:**

Our descriptive study revealed that the histological findings and amounts of mycobacterial DNA differed according to the sites despite being found in one lesion. Our results showed that the need for surgical debridement in each site was correlated with histological necrosis without inflammatory cell infiltration, as the inflammation is supposed to represent an active host immune response rather than mycobacterial burden. We suggest that the debridement of lesions with histological necrosis in mapping biopsy may be useful for Japanese cases with unsuccessful standard antibiotic treatment to achieve sufficient clinical improvement.

## Author summary

We have proposed a preoperative mapping biopsy procedure to perform optimal debridement for Buruli ulcer presenting complicated skin lesions. Briefly, multiple punch biopsies are performed from various sites around the Buruli ulcer lesions and the range of resection is decided according to the histological findings of the biopsies. Herein, we performed histological examination and quantitative PCR analysis using 83 tissue samples taken from mapping biopsy and debrided Buruli ulcer to validate our method. Our results suggested that the sites with histological necrosis need to be resected during surgical debridement and that the sites without histological necrosis can be preserved. These results in combination with those of previous studies are supportive of the mapping biopsy procedure that we have proposed. However, a randomized controlled study questioned the need for adjunct surgical treatment in Buruli ulcer. Further studies are needed to establish the Japanese evidence for surgical treatment in Japanese cases of Buruli ulcer.

## Introduction

Buruli ulcer is an ulcerative skin disease caused by *Mycobacterium ulcerans* infection. It has been reported in more than 33 countries including Japan [1]. Japanese cases are caused by *M*. *ulcerans* subspecies *shinshuense* [2]. Although the number of reported cases of Buruli ulcer in Japan has been increasing, delayed diagnosis is frequent [3–6]. The lesions occur commonly on the exposed area of the skin. Surgical treatment is performed in advanced cases in Japan in spite of the “destructive” cosmetic and functional complications caused by extensive debridement of large skin lesions on the exposed area, genitals, and breasts [7]. In addition, the paradoxical reaction, which results from antibiotic treatment [7, 8], makes it more difficult to determine the necessary and sufficient extent for resection. This phenomenon represents the paradoxical worsening of Buruli ulcer lesions, which is observed after starting antibiotic treatment. It may occur during or long after antibiotic treatment. In the past, it might have been mistakenly diagnosed as recurrence or relapse [1].

We have proposed a mapping biopsy procedure to perform adequate debridement for severe cases [3, 4, 9]. In these previous reports, multiple punch biopsies were performed from various sites around the Buruli ulcer lesions for mapping biopsy. We demonstrated that whether each site needs to be resected or preserved depends mainly on the histological findings not on the presence of mycobacteria or their DNA. We recommend that the sites with histologically massive necrosis must be resected during debridement and that the sites with histological inflammatory cell infiltration without significant necrosis can be preserved. The findings can be explained by mycolactone which is produced by *M*. *ulcerans*. Mycolactone is an immunosuppressive toxin that causes massive necrosis of the dermis and subcutis without inflammatory cell infiltration, which is the most characteristic histological feature of Buruli ulcer.

For this study, we hypothesized that *M*. *ulcerans* proliferating in the early phase of infection produces mycolactone to form less inflammatory necrotic lesions, whereas the decreased number of mycobacteria producing the toxin causes a host immune response in the skin [1, 10, 11]. In this study, we aimed to investigate the relationship between mycobacterial burden and histopathological findings reflecting the host immune response and then to evaluate tissue damage properly using our mapping biopsy procedure. We performed quantitative polymerase chain reaction (PCR) analysis using our mapping biopsy samples in combination with histopathological examination at each site.

## Methods

### Subjects

**Fig 1:**
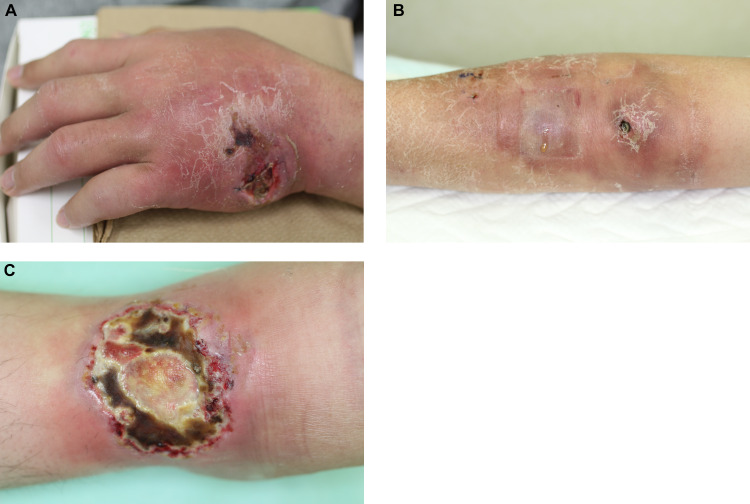
Clinical presentation of the four cases. (A–C) Clinical appearance of Cases 1–3, respectively. (A and B) Necrotic ulcers. Wide edema and erythema affected the hand and forearm. (C) Oval necrotic ulcers with halo.

Three Japanese patients with Buruli ulcer treated at Shiga University of Medical Science from 2011 to 2016 were included in this descriptive study. The diagnosis of Buruli ulcer was based on the compatible cutaneous manifestations, compatible histological findings, and positive IS*2404* amplification by PCR using DNA extracted from their skin tissue. Details of these cases were reported previously [3, 4, 9]. The clinical appearances of the cases are shown in Fig 1A to 1C.

### Tissue samples for mycobacterial tests and histological examination

The first skin samples were taken from the edge of the ulcers for diagnosis and subjected to histological examination with hematoxylin and eosin staining, and Ziehl-Neelsen staining, DNA extraction, and mycobacterial culture tests using Ogawa egg medium. DNA-DNA hybridization analysis using colorimetric microplate DNA-DNA hybridization ‘Kyokutou’ (Kyokutou, Tokyo, Japan) was performed in Case 1, in which the mycobacterial culture was successful.

After the diagnosis was confirmed, mapping biopsy procedures were performed. In the case of oval ulcer, tissue samples were taken from the sites 1 to 2 cm away in some directions from the edge of the ulcer. In Case 3, 12 samples from six directions were taken. In Case 1, the first mapping biopsy was performed to take four samples from sites 1–4 cm distant from the edge of the ulcer. In Case 2, 10 samples were taken from sites 1–10 cm away from the necrotic lesion. These procedures were performed to decide the ranges where debridement is required. In complicated lesions composed of edematous erythema and necrotic ulcers, tissue samples were taken from arbitrary sites on and around the gross lesions to determine the extent of debridement. In Case 2, 10 samples were taken from sites located around the edematous erythema lesion, not on the necrotic lesion. This was done to decide the range of resection. In Case 1, the first mapping biopsy revealed that histological necrosis was distributed widely throughout the dorsum of the hand. Thus, the second mapping biopsy was performed, and seven samples were taken from the edge of the edema or erythema, which were located in the palm and fingers.

In addition, a total of 84 tissue samples taken during debridement were analyzed. All tissue samples obtained in mapping biopsies were resected as deeply as possible using a 4-mm punch. The tissue samples were subjected to histological examination and DNA extraction as described above. The resected skin in surgical debridement was also subjected to mycobacterial tests and histological examination.

### PCR analysis

DNA extraction was performed using DNeasy Blood and Tissue kit (Qiagen, Tokyo, Japan) according to the manufacturer’s protocol. For the confirmation of the diagnosis, conventional PCR targeting IS*2404* (Genbank Accession number: KM459600.1) was performed in all cases as described elsewhere [12]. Quantitative real-time PCR was performed using DNA extracted from 29 samples taken from mapping biopsy to determine the quantities of IS*2404* in each skin sample and using the same primer pair as the conventional PCR and LightCycler 480 System II (Roche, Basel, Switzerland). The Cp values were determined by the second derivative maximum method and normalized using human glyceraldehyde 3-phosphate dehydrogenase (*GAPDH*) to correct the difference due to the cell density of punch biopsy skin specimens. The results of the quantitative PCR were examined descriptively in combination with the results of mycobacterial tests and histological findings. Specific statistical analyses were not performed due to the limited sample size.

### Ethics statement

This study was approved by the Medical Ethics Committee of Shiga University of Medical Science (approval number 30–103). We obtained written informed consent from all participants of this study.

## Results

### Subjects

The clinical features of our cases are shown in Table 1. All of them had ulcerative lesions on their extremities, two cases showed edematous erythema widely distributed on their hands and forearms with necrotic ulcers, and the other case showed oval ulcers with mild marginal erythema. No cases had past histories of immunosuppressive diseases. Two cases had coexisting methicillin-resistant *Staphylococcus aureus* infection.

**Table 1:** Clinical features of three cases of Buruli ulcer.

Cases	1	2	3
**Age (years)**	19	26	33
**Sex**	Male	Male	Male
**Affected sites**	Dorsum of the left hand	Left forearm and the elbow	Dorsum of the right foot
**Immunocompromised states**	No	No	No
**Clinical manifestation**	Edematous erythema with necrotic ulcer	Edematous erythema with necrotic ulcer	Oval ulcer
**Bacterial culture tests**	Methicillin-resistant *S*. *aureus*	Negative	Methicillin-resistant *S*. *aureus*
**Empirical** **non-standard** **antibiotic treatment**	Yes	Yes	Yes
**Surgical treatment**	Twice	Twice	Once

All patients received empirical non-standard antibiotic treatment before they were accurately diagnosed with Buruli ulcer. The combination of 800 mg/day of clarithromycin, 500 mg/day of levofloxacin, and 450 mg/day of rifampicin is recognized as the standard antibiotic treatment against *M*. *ulcerans* in Japan [13]. Cefdinir, faropenem, ciprofloxacin, and minomycin were used in Case 1; cefdinir, azithromycin, and minomycin were used in Case 2; and cefcapene was used in Case 3. These drugs are not included in the Japanese standard regimen of antibiotic treatment. Surgical debridement was performed in all cases as significant clinical improvement was not achieved after starting standard chemotherapy and the patients could not endure pain and distress. The lesions measured 5–15 cm in diameter. Surgical debridement was performed twice in Case 1, twice in Case 2, and once in Case 3. Debridement was performed based on our hypothesis that we should resect the sites with histological necrosis and preserve the sites without histological necrosis, i.e., with histological inflammation alone. All cases were cured after antibiotic and surgical treatments. We had to perform the second debridement in cases showing clinically overt gross necrosis. In the other two cases, obvious clinical evidence of paradoxical reaction was not observed.

### Mycobacterial tests

The summary of mycobacterial tests is shown in the supplementary information.

In Cases 1 and 3, smear tests were positive in the initial biopsy specimens and in the samples taken from the subcutis during debridement. In Case 2, the smear test was negative in the initial biopsy specimens. Smear tests using the subcutis sample taken during debridement were positive. Ziehl-Neelsen staining was also positive in the initial biopsy specimens and in the subcutis sample taken during debridement. In Case 1, mycobacterial culture tests using fresh lesional samples revealed growth of yellow-white colonies in the Ogawa egg medium. The skin samples were taken on the first visit (8 weeks after onset) and on the first debridement (10 weeks after onset), and the mycobacterial growth was observed 4 and 7 weeks, respectively, after the cultures started. DNA-DNA hybridization was positive for *M*. *marinum*. In the other two cases, mycobacterial growth was not observed.

### Histological examination

Histological examination was performed using 83 of the 84 tissue samples (56 from punch biopsies and 28 from surgical debridement). One sample was subjected to DNA extraction and mycobacterial tests without histological examination. Details of the samples are described in the supplementary information.

**Fig 2:**
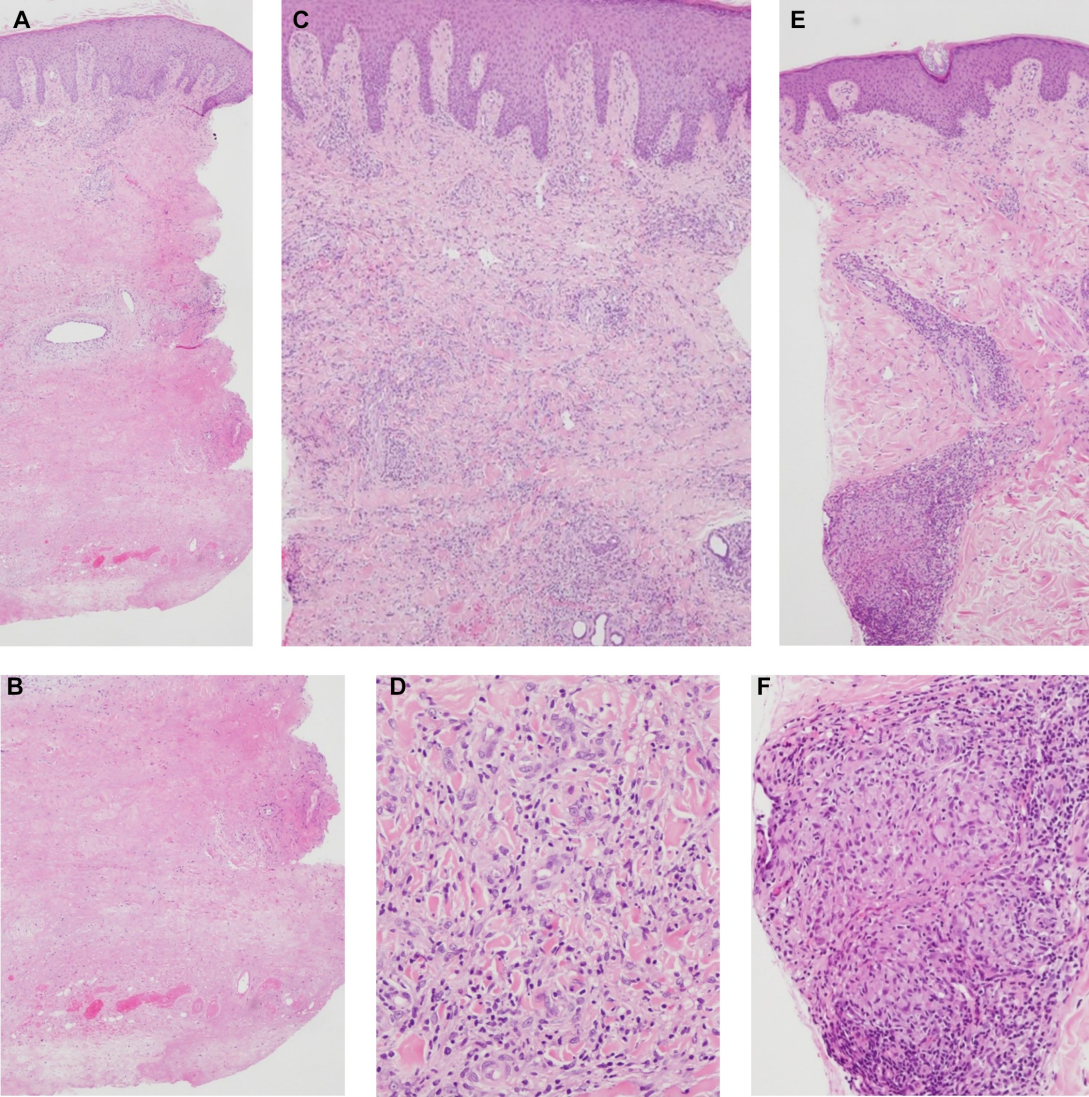
Representative histological findings of punch biopsies. (A and B) Sample 68 of Case 3 showed massive necrosis widely distributed in the deep dermis and subcutis. Epidermis and superficial dermis were nearly intact, except for the unspecific perivascular inflammatory cell infiltration in the superficial dermis. Necrosis was accompanied with scattered inflammatory cells and thrombus. This finding was recognized as necrosis (+), infiltrate (-), and granuloma (-) in the supplementary information. Hematoxylin and eosin staining, original magnification, ×40 (A) and × 100 (B). (C and D) Sample 67 of Case 3 showed dense inflammatory cell infiltration distributed over the entire dermis. Necrosis was not observed in this sample. (D) Inflammatory cells were mainly composed of lymphocytes and histiocytes. This finding was recognized as necrosis (-), infiltrate (+), and granuloma (-) in the supplementary information. Hematoxylin and eosin staining, original magnification, ×100 (C) and ×200 (D). (E and F) Sample 71 of Case 3 showed mild inflammatory cell infiltration and granuloma formation in the dermis. This finding was recognized as necrosis (-), infiltrate (+), and granuloma (+) in the supplementary information. Hematoxylin and eosin staining, original magnification, ×40 (E) and ×200 (F).

In this study, we divided the histological findings into three patterns, that is, massive necrosis, dense inflammatory cell infiltration, and granuloma formation. The representative histological pictures of each pattern are shown in Fig 2A to 2F. In this respect, we described the results of the histological examination in the supplementary information.

Inflammatory cell infiltration was the most frequent finding in our study. Of the 83 samples, 73 showed histological inflammation. Massive necrosis was observed in 45 samples. A coexistence of necrosis and inflammation was observed in 35 samples. Therefore, 10 samples showed histological necrosis without inflammation and 38 samples showed inflammation without necrosis. Granuloma formation was always accompanied with inflammation. A coexistence of necrosis and granuloma formation was also observed. Necrosis was observed in the sites near the center of the lesions or in the samples taken from debridement. Moreover, 43 of 83 samples were taken in mapping biopsies. As a result, necrosis alone, inflammation alone, and their coexistence were observed in 2, 26, and 15 samples, respectively. Debridement was performed so that the sites with histological necrosis were resected based on our hypothesis.

**Fig 3:**
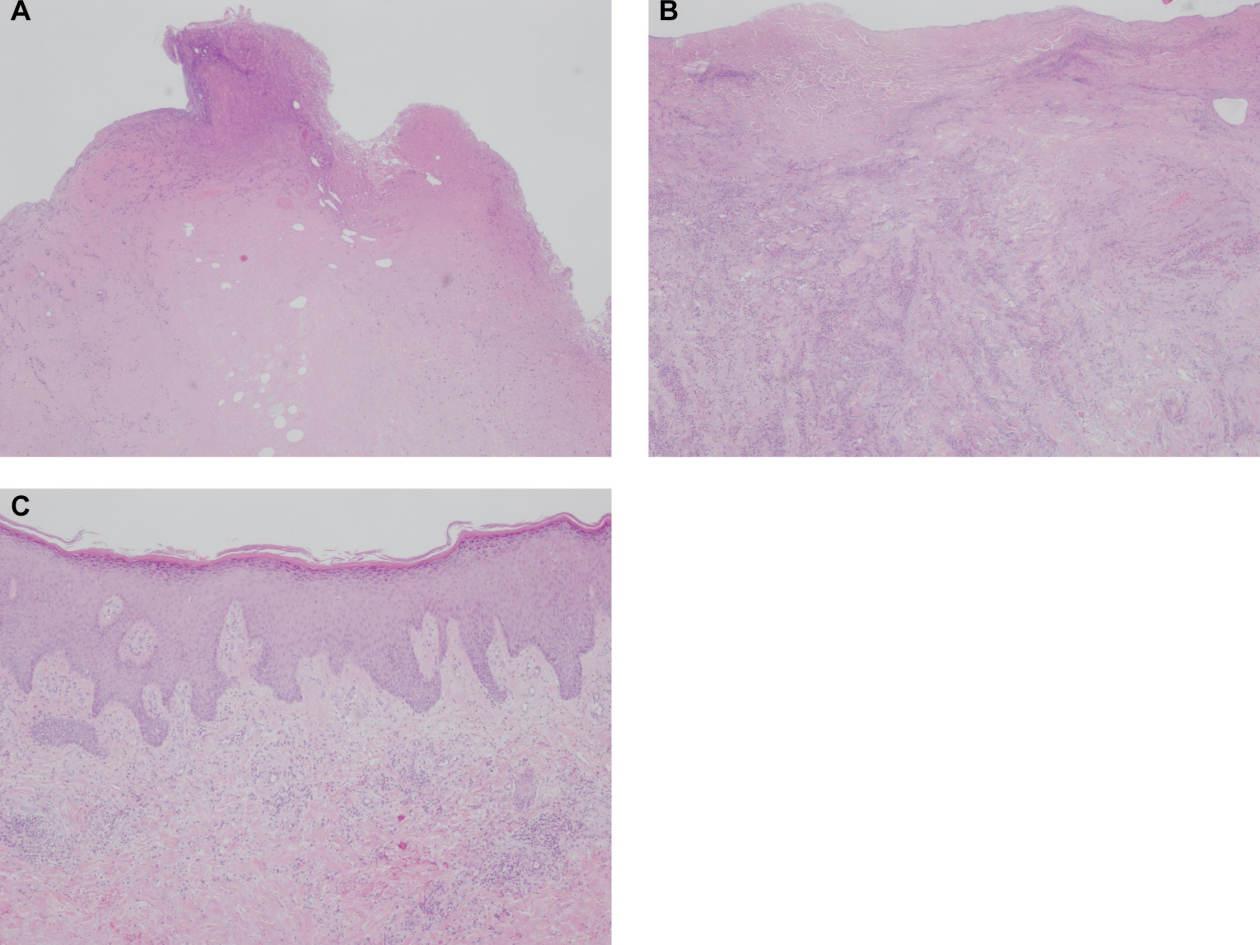
Histological findings of the skin resected during debridement. (A) Histology of the center of the ulcer. Massive necrosis forming the necrotic core was observed. (B) Histology of the adjacent lesion of the ulcer. The epidermis was also gone, and dense inflammatory cell infiltration was observed on the background of necrosis. This showed the infiltration belt surrounding the necrotic core. (C) Histology of the site 1 cm away from the ulcer. Epidermis was almost intact, except for the finding of acanthosis. Inflammatory cell infiltration was mild compared to (B) and necrosis was not observed. This site was located outside of the infiltration belt. Hematoxylin and eosin staining, original magnification, ×100.

In Case 3, histological specimens of tissue samples in debridement were cut so that we could evaluate them outwardly from the center of the lesion (or the ulcer) to the margin of the lesion. The representative picture is shown in Fig 3A to 3C. Massive necrosis was observed in the center of the lesion. Adjacent to the necrosis, dense inflammatory cell infiltration was observed. Necrosis and hemorrhage coexisted with the infiltration. On the outside, the healthy tissue gradually appeared.

In 34 sites, histological examination was performed more than once to mainly evaluate the histological changes over time and to resect during debridement the sites where punch biopsy was once performed. For example, Sample 9 in Case 1 was histologically examined once in punch biopsy and was then resected during debridement followed by the second histological examination as Sample 18. The observation of such sites revealed that no sites with histological necrosis showed the disappearance of necrosis over time or after the administration of various antibiotics. In addition, we found no tendency for the histological necrosis to decrease in samples taken even in the late phase of the disease or after antibiotic administration. In contrast, in the 11 biopsied sites where histological necrosis was not observed, ulcers were cured and no recurrence was observed even without resecting these sites.

### Quantitative PCR

**Fig 4:**
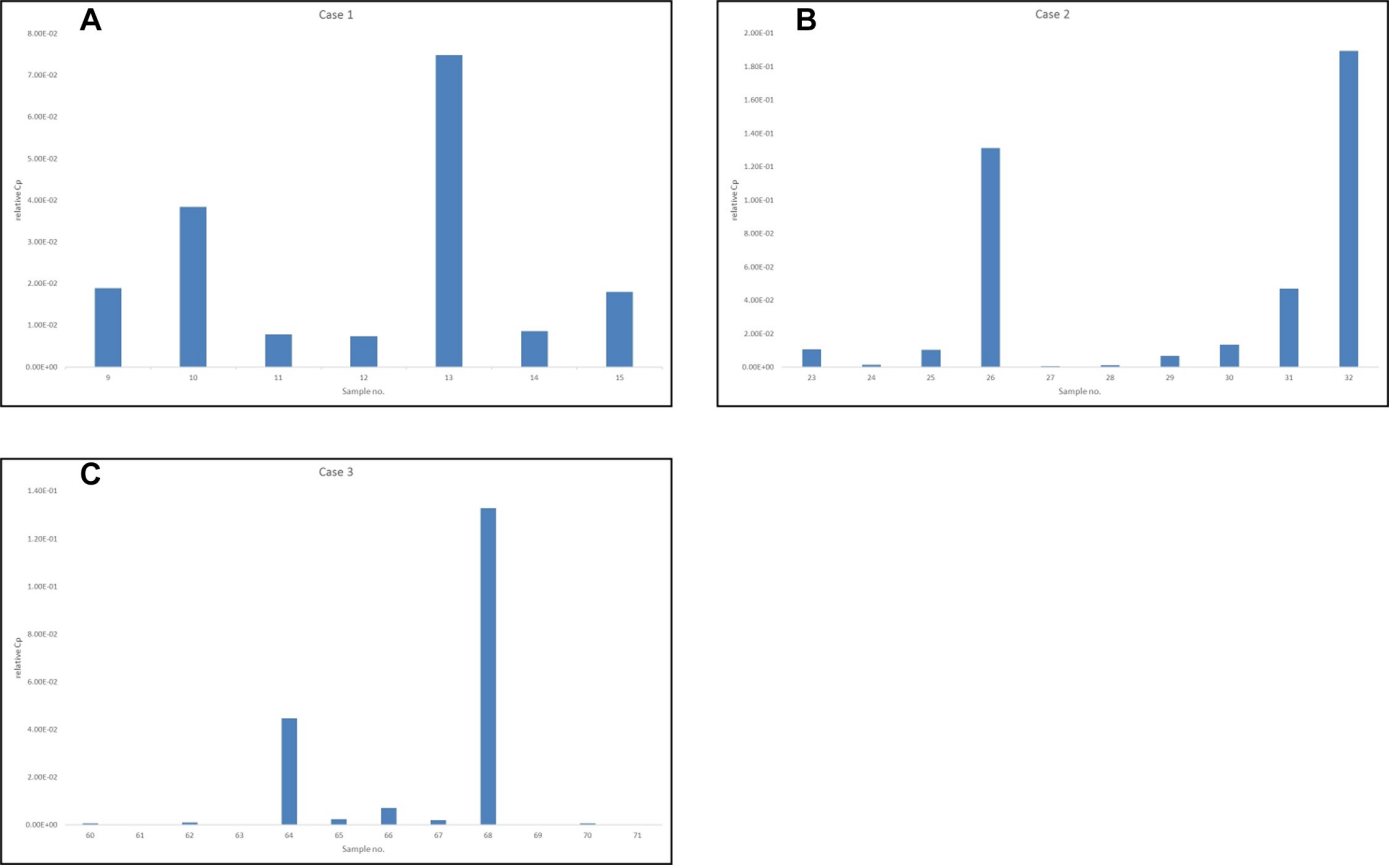
Bar charts of the results of the quantitative PCR in Cases 1 to 3. The Y axis shows the relative Cp values normalized with human *GAPDH*. GAPDH, glyceraldehyde 3-phosphate dehydrogenase; PCR, polymerase chain reaction.

The results of the quantitative PCR are shown in bar charts (Fig 4A to 4C). Sites with low Cp values in the quantitative PCR tended to show necrosis in the histological examination. Notably, in Case 3, Sites 64 and 68, where histological necrosis without inflammation was observed, showed obviously lower Cp values than other sites.

## Discussion

In our previous studies [3, 4, 9], we have tried to examine the relationship between mycobacteria and histological changes in Buruli ulcer lesions based on only whether IS*2404* amplification was detected by conventional PCR. In the present study, we performed quantitative PCR for IS*2404* using DNA extracted from skin samples taken in our mapping biopsies. Quantitative PCR for IS*2404* using human samples was reported previously [14, 15]. Swab samples were used, and a good correlation was demonstrated between the amount of *M*. *ulcerans* DNA in extracts from swabs and the positivity of direct smear microscopy. As a result, the Cp values were substantially different according to the sites. This result more clearly illustrated an uneven distribution of mycobacterial DNA in the lesions compared to our previous results.

In the histopathology of Buruli ulcer, massive necrosis in the deep dermis and subcutis with scant and unnoticeable inflammatory cell infiltration is well known [1, 16]. Dense inflammatory cell infiltration mainly composed of lymphocytes and histiocytes is also frequently observed. Infiltration around the sweat glands is often noted. This histological type is often accompanied with necrosis and granuloma formation. As for the histological examination to avoid unnecessary radical surgical resection, we have proposed performing the mapping biopsy procedure before debridement, especially for cases with affected exposed area and joint sites where performing extensive debridement is difficult [3, 4, 9]. As pointed out in our previous study [9], the center of the lesions showed massive necrosis, and adjacent to it, dense inflammatory cell infiltration was observed. On the outside, densely infiltrated tissue transitions to the healthy skin. Granulomas seemed to be formed later after inflammatory cell infiltration.

Our proposal in previous studies is that the sites where necrosis is noted in mapping biopsy needs to be resected and that those that showed inflammatory cell infiltration without histological necrosis will be preserved. Ruf et al. who first described these histological characteristics [8, 11, 16] called the central necrotic lesions as the necrotic core and the dense inflammatory cell infiltration around the necrosis as the infiltration belt [8]. Our mapping biopsy, in other words, intends to find the location of the infiltration belt and to resect the necrotic core. Therefore, we think that our method corresponds to the findings of another previous study [3, 4, 9].

In our histological evaluation of 84 sites, 34 sites were evaluated histologically more than once. Ruf et al. described that a partial infiltration of the affected tissue was observed after antibiotic treatment, whereas a damaged subcutis with extensive necrosis without major signs of infiltration was observed before treatment [16]. However, as they have illustrated, the histological difference resulted not from the simple effects of antibiotic treatment or the changes over time but from the conflict between mycobacteria with immunosuppressive toxin and host immune response [8]. As to this point, two studies also illustrated the histological changes after antibiotic treatment [17, 18]. They described that highly organized lymphoid structures or bands of inflammatory leucocytes were observed in Buruli ulcer patients after the completion of the standard antibiotic treatment. They suggested that the histological changes indicated the healing process and that such changes developed at the margins of the lesions. Based on their conclusions, our method may be useful to clarify the border between the residual necrotic lesions and the healing sites after antibiotic treatment. In addition, our results suggested that inflammatory infiltration was formed before the completion of the standard antibiotic treatment. Antibiotics administered before an accurate diagnosis, which is not recognized as standard, may be also effective for mycobacteria.

Our histological examination based on mapping biopsies in combination with quantitative PCR revealed a tendency that the Cp values were relatively lower in the sites with histological necrosis than in the sites without necrosis. Especially, in Case 3, the Cp values were significantly lower in sites where necrosis without structured inflammatory cell infiltration was the major histological finding than those in sites with histological inflammation. However, in Case 1, Samples 13 and 15 showed relatively low Cp values, although they did not have histological necrosis and could be preserved. We think that these results support the notion that large amounts of immunosuppressive toxin produced by a lot of mycobacteria caused the histological massive necrosis without inflammation and that decreased mycobacteria and toxin led to the reconstitution of the host immune response. Therefore, we speculated that the sites showing evidence that host immune response will work could be preserved even if mycobacteria and their DNA are detected.

Based on our observations, we would like to emphasize the following three points. First, none of the 34 sites described above showed the disappearance of histological necrosis. Ruf et al. also described that large necrotic areas remained unchanged in spite of the completion of the antibiotic treatment [16] and that the mycobacteria cannot be reached by the infiltrating cells around the necrosis [8]. We speculated that the antibiotics cannot reach the necrotic core without blood flow and that the necrotic core must be resected once it was widely formed. Second, none of the 11 sites that showed histologically inflammatory cell infiltration needed to be resected to cure the ulcers. The result suggests that if the necrotic core inside is sufficiently removed, the sites with the infiltration belt can be preserved. Here, the host immune response and antibiotics can eliminate the mycobacteria. However, a histological finding of dermal infiltration and subcutaneous necrosis is often observed. Sites showing this histological pattern may need debridement, as they contain a part of the necrotic core to be resected. Finally, the conflict between mycobacteria and host immune response, which is reflected as the difference in histological findings, will vary in each site even if the biopsies are performed from one patient at the same time. For example, in the mapping biopsy of Case 3, Samples 64 and 68, which were taken at 1 cm away from the edge of the ulcers, showed significant necrosis. In contrast, Samples 60, 62, 66, and 70, which were also taken at sites 1 cm away, showed inflammatory cell infiltration with granuloma formation. This can be understood considering the existence of the infiltration belt; however, for example, in Samples 9–15, various sites on the left hand showed histologically inflammatory cell infiltration without necrosis, whereas the site on the wrist (Sample 10) only showed necrosis regardless of the distance from the center of the lesion. In such complicated cases, the obvious location of the single infiltration belt cannot be determined. This suggests that the conflict between mycobacteria and host immune response is not a single event but occurs everywhere around the lesion. We speculate that host immune response on one site may fail to eliminate the mycobacteria to form necrotic lesions, whereas host immune response on the other site prevents the spread of mycobacteria. The latter will show an inflammatory cutaneous manifestation, which presents clinically, for example, as an edematous erythema or a paradoxical reaction. Complicated lesions such as Cases 1 and 2 might be formed in this manner.

As regards the surgical intervention for cases with Buruli ulcer, a randomized controlled study was performed, which showed that delaying the decision to perform surgery did not delay the healing rate and increase residual functional limitations [19]. Moreover, even large ulcers can heal without surgical intervention. Therefore, our decision at the time, i.e., whether each site needs debridement or not, was based on the assumption derived from the analysis of the biopsy, not on the clinical evidence. We speculate that the results of the clinical trial in an African setting may not completely be applicable to Japanese cases, as surgical treatment is more accessible and the cosmetic results are highly appreciated in Japan. Clinical evidence of the surgical treatment for Japanese cases with Buruli ulcer is scarce due to the rarity of the disease. In Japanese cases, surgical treatment is recommended especially when the diagnosis is delayed [13, 20]. Our cases were accurately diagnosed after months of evaluation. The first biopsies were performed from 6 to 13 weeks after onset. Inflammatory cell infiltration was histologically recognized first in the samples taken 10, 6, and 13 weeks after onset respectively in three cases. Therefore, we suggested that the host immune response may begin from this period. However, antibiotic treatment alone failed to achieve obvious improvement in our three cases, and we decided to perform debridement.

This study has some limitations. First, Japanese cases with Buruli ulcer are extremely rare. Therefore, the sample size of the current study is small and the study is based on a Japanese setting. Further investigation is required to validate the efficiency of our histological mapping method. As described above, in an African setting, a randomized controlled study questioned the need for adjunct surgical debridement in treating Buruli ulcer. Hereafter, the establishment of clinical evidence for the surgical treatment of Japanese Buruli ulcer cases is required. Moreover, our mapping biopsy is based on the Japanese situation in which histological services are relatively accessible and histological examination results are obtained in a week. Second, the spatial distribution of *M*. *ulcerans* in skin tissue is uneven [15]. They are known to be located primarily in the subcutis. The sizes of our skin samples, in other words the sizes of the resected subcutis, may have some influence on the results of the quantitative PCR. To avoid such influence, we tried to correct the Cp values using human *GAPDH*. However, we must recognize that punch biopsies may not always be helpful as mycobacteria are not evenly distributed.

**Fig 5:**
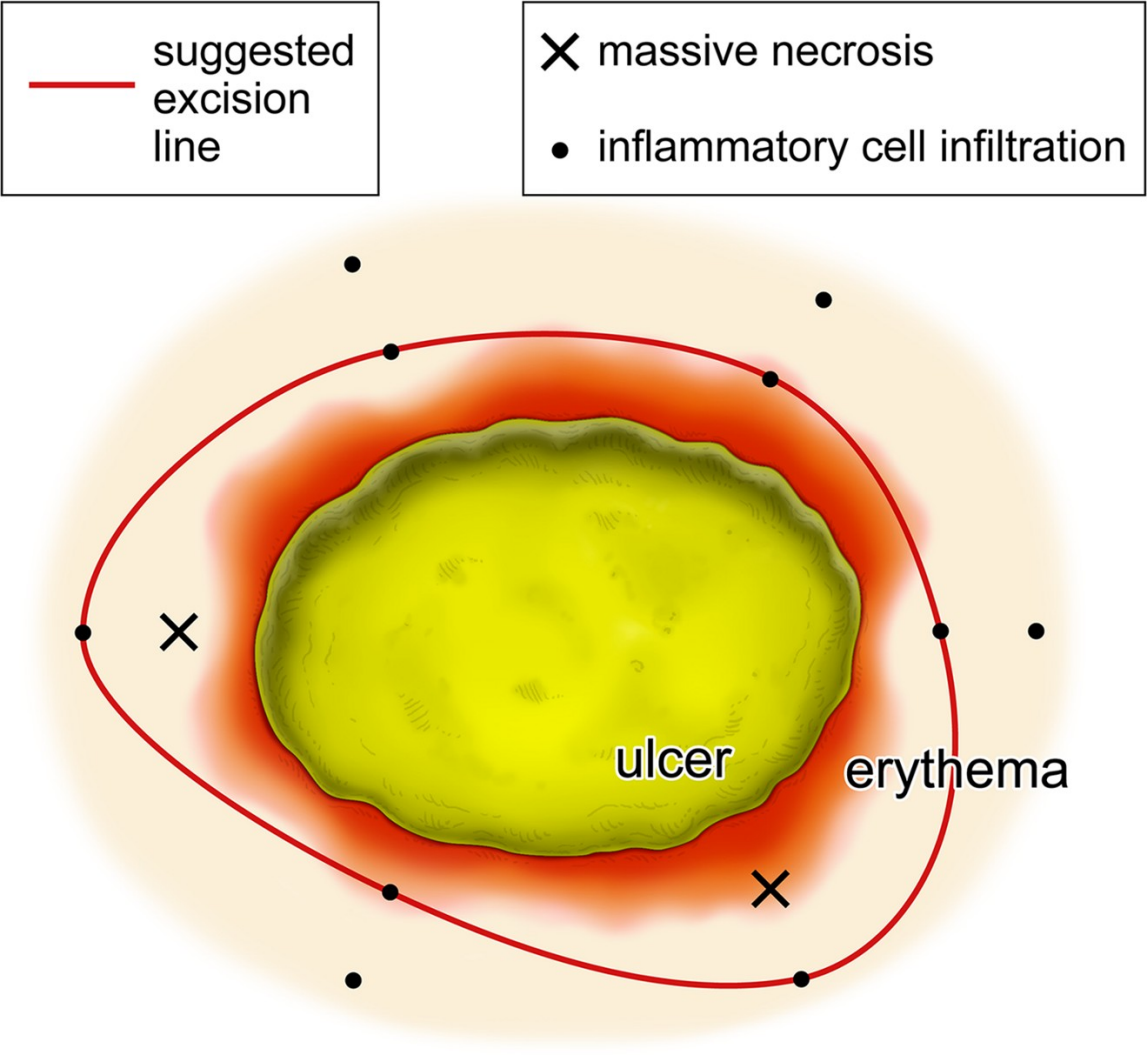
Outline of our mapping biopsy method.

In this study, we performed descriptive analysis of the histological findings and amounts of mycobacterial DNA in each site of Buruli ulcer lesions. In conclusion, the amounts of mycobacterial DNA were shown to differ substantially according to the sites even though they belonged to one lesion. Our study also showed that the histological findings differed significantly even though the samples were taken simultaneously from nearby sites. The results in combination with previous studies are supportive of our mapping biopsy procedure. Multiple skin biopsies will provide the information on the location of the necrotic core and infiltration belt. Based on the results, the necrotic core must be resected during surgical debridement, but it can be preserved outside of the infiltration belt. Fig 5 illustrates the outline of our method. We believe that the method is preferable in particular for cases with complicated lesions that are composed of edematous erythema and necrotic ulcer, and performing extensive surgical debridement is difficult due to cosmetic and functional reasons.

## Supporting information

S1 Table Summary of the analysis of 84 tissue samples. The results of mycobacterial and histological examination and PCR analysis are shown.
(XLSX)
